# White matter fractional anisotropy is related to processing speed in metabolic syndrome patients: a case-control study

**DOI:** 10.1186/1471-2377-10-64

**Published:** 2010-07-27

**Authors:** Bàrbara Segura, María Ángeles Jurado, Núria Freixenet, Núria Bargalló, Carme Junqué, Adrià Arboix

**Affiliations:** 1 Department of Psychiatry and Clinical Psychobiology. Faculty of Psychology. University of Barcelona. Barcelona. Spain; 2 Institute for Brain, Cognition and Behavior (IR3C). Faculty of Psychology. University of Barcelona. Barcelona. Spain; 3 Diabetes, Endocrinology and Nutrition Service. Hospital de Sabadell. Corporació Sanitària Parc Taulí. Barcelona. Spain; 4 Centre de Diagnòstic per la Imatge Hospital Clínic de Barcelona (CDIC), Hospital Clínic de Barcelona. Barcelona. Spain; 5 Institute of Biomedical Research August Pi i Sunyer (IDIBAPS), Faculty of Medicine. University of Barcelona. Barcelona. Spain; 6 Cerebrovascular Division, Department of Neurology, Hospital Universitari del Sagrat Cor, University of Barcelona. Barcelona. Spain

## Abstract

**Background:**

Metabolic Syndrome (MetSd) is a cluster of vascular risk factors that may influence cerebrovascular pathology during aging. Recently, microstructural white matter (WM) changes detected by diffusion tensor imaging (DTI) and processing speed deficits have been reported in MetSd patients. We aimed to test the relationship between WM alteration and cognitive impairment in these patients.

**Methods:**

The sample comprised 38 subjects (19 patients aged between 50 and 80 years old, and 19 controls). All patients fulfilled National Cholesterol Education Program Adult Treatment Panel III (NCEP ATP-III) criteria for MetSd. Speed of information processing was measured by the Symbol Digit Modalities Test (SDMT) and reaction time (RT) on the Continuous Performance Test (CPT-II) and the Grooved Pegboard Test (GPT). DTI images were acquired in a 3 Tesla Siemens Trio scanner. Voxelwise statistical analysis of the fractional anisotropy (FA) data was performed using the Tract-Based Spatial Statistics part of the FMRIB Software Library. A correlation analysis was performed between processing speed variables and FA values.

**Results:**

There was a larger proportion of slow subjects (percentile below 25^th^) in the patient group (Chi^2 ^= 7.125 p = 0.008). FA values correlated positively with SDMT in anterior and posterior parts of the corpus callosum, and RT CPT-II correlated negatively with FA values in the anterior corpus callosum (p < 0.05 corrected) in the patient group.

**Conclusion:**

We found significant correlations between WM alterations and cognitive impairment in MetSd patients, especially in the frontal lobe. These findings highlight the importance of MetSd prevention and control due to its association with structural and functional damage in the central nervous system.

## Background

Metabolic Syndrome (MetSd) is a cluster of risk factors including hypertension, hyperglycemia, dyslipidemia, and central obesity associated with cardiovascular disease. The prevalence of MetSd is increasing in modern-day societies and the condition is now very common during aging [1].

Recent DTI studies have shown region-specific patterns of WM alterations, such as myelin loss and axon degeneration in humans [2,3] and animals [4,5].

Among the DTI indexes, FA has been defined as a measure of tract directionality and integrity. Decreases in FA have been observed in association with aging [6], especially in the frontal lobe [7]. Moreover, FA decrease has been related with changes in other diffusion measures such as axial diffusivity (AD), radial diffusivity (RD) and mean diffusivity (MD). The specific combinations of these variables are related to microstructural deterioration mechanisms in ageing. For instance chronic WM degeneration, demyelination, subtle microstructural alterations, secondary Wallerian degeneration and gliosis or early axonal injury [2].

In spite of the increasing importance of MetSd in the aging process, the association of the condition with brain damage and cognitive performance has not been widely studied.

Previous magnetic resonance imaging (MRI) studies report some evidence of the effect of MetSd on the brain, especially in WM. These studies describe the presence of periventricular hyperintensities, subcortical WM lesions [8] and silent lacunar infarcts [9] in MetSd samples. In a recent DTI study [10] of MetSd patients, we found an anterior-posterior pattern of deterioration in WM with reduced fractional anisotropy (FA) and increased apparent diffusion coefficient (ADC) values.

The presence of microangiopathies is frequent in the elderly and constitutes an independent risk factor for recurrent vascular events and cognitive impairment. Today, there is growing interest in the study of the early phases of small vessel disease, and the follow-up of prodromal states of pathology may help to identify the factors that influence the progression.

Moreover, some authors associate MetSd in aging with high risk of dementia [11], fronto-subcortical symptoms [12,13] and poorer neuropsychological performance [14,15]. In a recent study, we found differences between MetSd and control groups in processing speed and in some executive functions, after controlling for the influences of education and gender[15]. Our results suggested that MetSd may be a prodromal state of vascular cognitive impairment. In recent studies, slower processing speed in MetSd patients is the most consistent finding [16], even when the subjects are relatively young [15].

To the best of our knowledge, the relation between WM alterations and neuropsychological profile in MetSd patients has not been studied to date.

Relationships between the loss of WM integrity and slowed processing speed have been shown in healthy people [17] and neurological disorders, and the importance of corpus callosum preservation in good signal transduction has been stressed [18]. Processing speed is a basic cognitive or brain processes that subserves many other higher-order cognitive domains. The speed with which an individual performs a cognitive activity is not simply a function of the processes required in that activity but also a reflection of his or her ability to carry out many different types of processing operations. Slowing with age is often considered one of the best-documented and least controversial behavioural phenomenon of aging [19]. As a basic process that is dependent on basic neuronal function and glial support, any sort of focal or diffuse injury to the brain can affect processing speed. In central nervous system oligodendrocytes are the glial cell responsible to myelin sheath formation. Myelin acts as insulator and it accelerates the neural signal through the axons, therefore it is crucial to modulate the speed of signal transduction [20]. When WM is disrupted then processing speed is in danger to be affected [18,21].

In this study, we hypothesize that MetSd slowness, evidenced by neuropsychological assessment, would be associated with subtle FA decreases. To test this possibility we performed a correlation study between FA and processing speed variables using DTI.

## Methods

### Subjects

The study included thirty-eight subjects from two public medical centers in Cerdanyola del Vallés and Sant Just Desvern in the province of Barcelona (Spain). The present sample participated in two previous works [10,15]

We recruited 19 MetSd and 19 controls. To be diagnosed with MetSd, participants had to fulfill at least 3 out of 5 criteria listed in Table 1 (NCEP criteria) [22]. The criterion for selecting controls was the absence of any vascular risk factors included in the MetSd criteria. All the participants were volunteers, right-handed, and aged between 50 and 80 years old. The exclusion criteria for both groups and details of the methods used have been explained elsewhere [2].

**Table 1:** Metabolic Syndrome criteria

Measure	Categorical Cut-off points
Elevated waist circumference	≥ 102 cm in men ≥ 88 cm in women
Elevated triglycerides	≥150 mg/dL or On drug treatment for elevated triglycerides
Reduced HDL-Cholesterol	< 40 mg/dL in men < 50 mg/dL in women or On drug treatment for reduced HDL-cholesterol.
Elevated blood pressure	≥ 130 mm Hg systolic blood pressure or ≥ 85 mm Hg diastolic blood pressure or On antihypertensive drug treatment.
Elevated fasting glucose	≥ 100 mg/dL or On drug treatment for elevated glucose.

HDL: High density lipoproteins

The sample comprised a patient group of eleven women and eight men with mean age 61.26 years (SD = 7.19) and mean years of education 10.37 (SD = 3.55), and a control group of eleven women and eight men with mean age 59.63 years (SD = 5.37) and mean years of education 11.68 (SD = 3.59).

All enrolled subjects gave written informed consent prior to taking part in the study, and the research was conducted in accordance with the Helsinki Declaration. The study was approved by the ethics committee of the University of Barcelona.

### Neuropsychological examination

All participants underwent a comprehensive neuropsychological examination performed by a trained neuropsychologist (B.S.). Intelligence quotient was estimated using Vocabulary subtest of Wechsler Adults Intelligence Scale-III.

Several criteria have been proposed to guide the selection of measures used to assess processing speed: relative simple task, the speed measure should not merely represent the input and output processes or sensory and motor processes, it is desirable that the construct be evaluated with several measures to minimize the specific variance associated with single measures and to emphasize common construct-relevant variance [19].

We selected processing speed test in base of their sensibility to processing speed changes in previous studies. The processing speed variables used to test our hypothesis were total correct responses in 90s on the orally-administered SDMT [23], and time taken to complete the GPT with dominant and non-dominant hands [23] and RT on the CPT-II[23].

All the selected tests provide measures of processing speed. Specifically, the oral SDMT test also involves divided attention, complex visual scanner tracking, perceptual speed and memory. The use of the oral version avoids motor influences in this processing speed measure. GPT involves eye-hand coordination and motor speed: some studies have shown slowed GPT execution in patients with vascular risk factors [24,25]. Finally, CPT-II assesses sustained attention to a visual-motor task and response inhibition. RT measures in this latter task show the mean response time to the targets [26], the computerized reaction time measures allow obtaining higher accuracy. All the variables were recorded as part of a more extensive neuropsychological assessment described elsewhere [15].

### MRI data acquisition

Data acquisition was performed on a 3 Tesla Siemens Magnetom Trio scanner (Erlangen, Germany), belonging to the *Institut d'Investigacions Biomèdiques August Pi i Sunyer *(IDIBAPS), at the Radiology Service of the Hospital Clínic of Barcelona. The DTI sequences were acquired with the following parameters: TR = 5533 ms, TE = 88 ms, acquisition matrix = 122 × 122, field of view (FOV) = 250 × 250 mm^2^, diffusion directions = 30, slice thickness = 2 mm, gap distance = 0.6 mm, number of slices = 44, b-values: 0 and 1000 s/mm^2^, IPAT factor = 2, total scan time 3:10 min.

### Image pre-processing

Voxelwise statistical analysis of the FA data was carried out using Tract-Based Spatial Statistics, [27], part of the FMRIB Software Library [28]. First, FA images were created by fitting a tensor model to the raw diffusion data using FDT, and then brain-extracted using BET [29]. FA data for all subjects were then aligned into a common space using the nonlinear registration tool FNIRT [30,31] which uses a b-spline representation of the registration warp field [32]. Next, the mean FA image was created and thinned to produce a mean FA skeleton (FA threshold to 0.2) which represents the centers of all tracts common to the group (control or patient). The aligned FA data for each subject were then projected onto this skeleton and the resulting data fed into voxelwise cross-subject statistics (permutation-based, 5000 permutations).

### Assessment of white matter hyperintensity (WMH) burden

The DTI sequence also provides a T2 weighted volume (B0) which was used to assess WMH. Briefly, a board-certified neuroradiologist (N.B) rated all images using the Fazekas scale [33]. The scale is labeled as 0 = absence, 1 = punctate foci, 2 = beginning confluent of foci, 3 = large confluent areas.

### Statistical analysis

For the analysis of the neuropsychological data, raw data were first transformed into percentile ranks (pc). The transformation of raw to percentile scores allows us to define the threshold which determines slow performance on cognitive tests in the study-sample (percentile = 25) in comparison to the normative population. This threshold allows us to detect the amount of subjects with scores in the fourth quartile of normal distribution.

A qualitative variable was created for each neuropsychological quantitative variable indicating the frequency of slow participants.

Demographic and neuropsychological variables and the presence of WMH in the groups were compared using the Statistical Package for the Social Sciences (SPSS WIN; v.14.0). The Mann Whitney U test was used for quantitative variables when required, while the χ^2 ^test was used to analyze qualitative variables. A p value < 0.05 was considered statistically significant.

Correlations between DTI values and processing speed variables were examined in the patient and control groups using the FMRIB software library's randomized TFCE (Threshold-Free Cluster Enhancement) option [34]. In our analyses we used default settings for TFCE and performed 5000 permutations. A standard general linear model design matrix was applied using raw scores. Results were reported at p values < 0.05, corrected. To control the possible effect of age within group correlations, we performed post-hoc partial correlation analysis between the mean FA of significant cluster and processing speed variable.

## Results

There were no differences between the groups in age, years of education, intelligence quotient estimation or gender (Table 2). All participants were from the same geographical area. There were no significant differences between groups in the period between psychological testing acquisition and the scan session (t = 1.27 p = 0.312). Mean time in days between both sessions was mean 75.95 (SD+81.02) in patients and mean 108.21 (S.D+110.40) in controls.

**Table 2:** Demographic data comparison between groups

	Metabolic Syndrome		Control			
	**Mean (SD)**	**Range**	**Mean (SD)**	**Range**	**U**	**p value**
Age (years)	61.26(7.19)	50-75	59.63(5.37)	51-67	160.5	0.563
Education (years)	10.37(3.55)	5-16	11.68(3.59)	8-16	138.5	0.223
Vocabulary	42.21(8.651)	28-58	42.89(9.427)	25-60	168.5	0.729

S.D: standard deviation, U: Mann Whitney U test.

The neuropsychological examination of all controls showed normal cognitive performance in comparison with standard population scores. This result ruled out the possibility of cognitive impairment in any of our subjects. Patients and controls showed WMH, typically described in older samples, but there were no differences between the rates of WHM in the two groups (Chi^2 ^= 2.182 p = 0.336).

Comparison of neuropsychological raw data did not reveal significant differences between groups, even in processing speed variables (data previously reported in [2]. However, SDMT and GPT ND identified significantly larger proportion of slow-performance subjects (pc < 25) in the patient group (Table 3). No significant differences were found comparing the other processing speed variables.

**Table 3:** Cognitive test results

	Metabolic Syndrome			Control				
**Cognitive Test**	**Mean(SD)**	**Range**	**Pc < 25**	**Mean(SD)**	**Range**	**Pc < 25**	**Chi**^**2**^	**p value**
SDMT	42.63 (15.90)	19-61	6	49.58 (11.57)	31-68	0	7.135	0.008
GPT DH	88.53 (37.88)	59-221	7	72.16 (12.81)	59-110	3	2.171	0.141
GPT NDH	99.74 (41.63)	62-233	8	81.95 (24.22)	55-170	2	4.886	0.027
CPT-II RT	440.53 (71.33)	329-557	8	422.01 (55.23)	322-487	7	0.110	0.740

SDMT = Symbol Digit Modalities Test; GPT = Grooved Pegboard Test, DH = Dominant Hand, NDH = Non Dominant Hand; CPT-II RT = Continuous Performance Test Reaction time; Pc = Percentile; Chi^2 ^= Chi square test.

**Figure 1:**
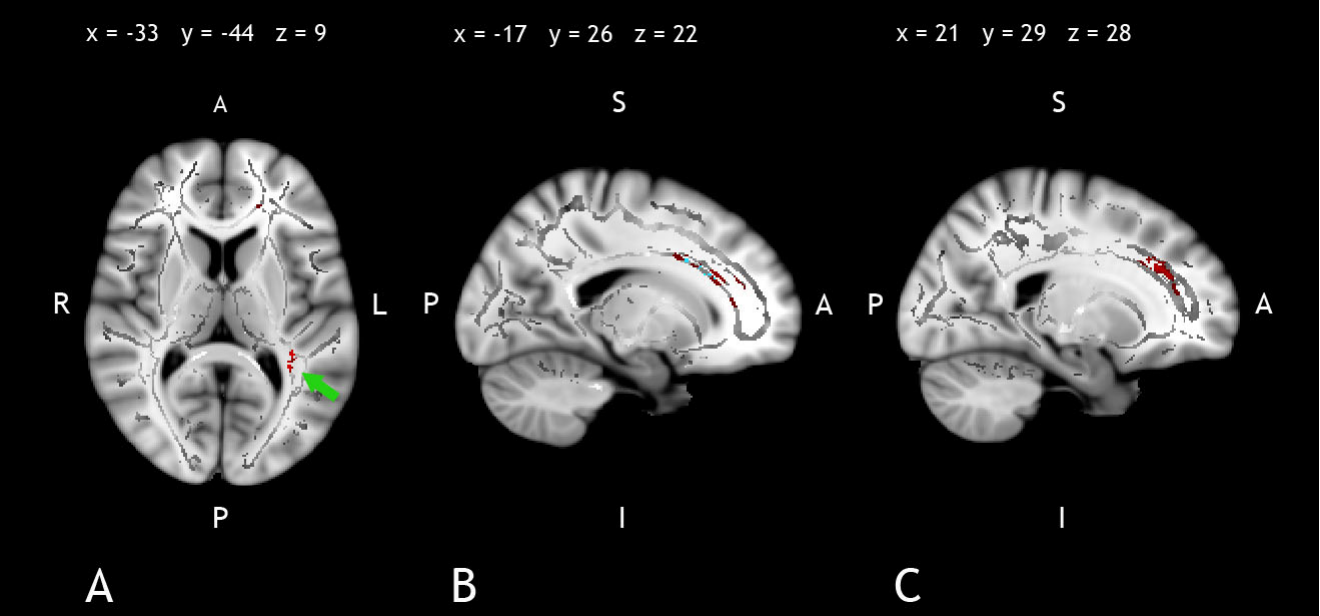
**Correlation Results between FA Values and Processing Speed Variables**. Figure 1.A, 1.B and 1.C show positive correlation (warm) between FA values and SDMT performance. Figure 1.B shows negative (cool) correlation between FA and RT CPT-II performance. Images are represented according to radiological convention (left corresponding to the right hemisphere). R: right, L: left, A: anterior, P: posterior, I: inferior; S: superior.

**Figure 2:**
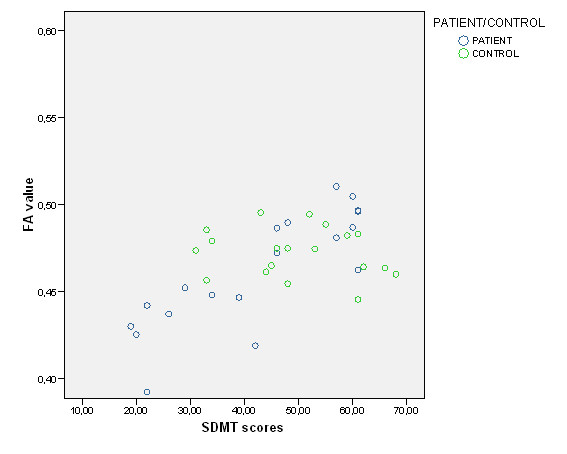
**FA values and SDMT scores (for both patients and controls) in 21, 28, 29 coordinate significant cluster**. Y axis FA value in 21, 28, 29 coordinate cluster. X axis SDMT scores.

**Figure 3:**
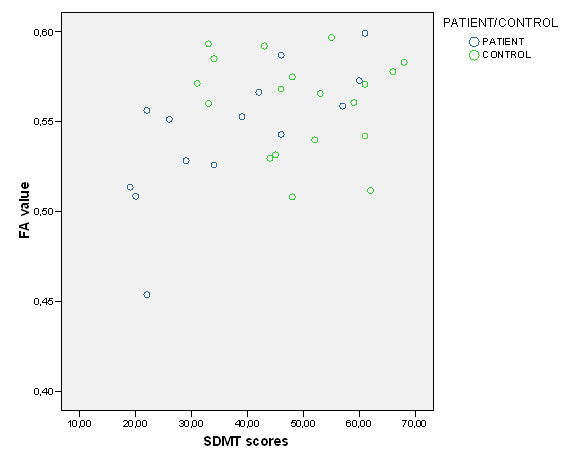
**FA values and SDMT scores (for both patients and controls) in -33, - 44, 9 coordinate significant cluster**. Y axis FA value in -33, -44, 9 coordinate significant cluster. X axis SDMT scores.

**Figure 4:**
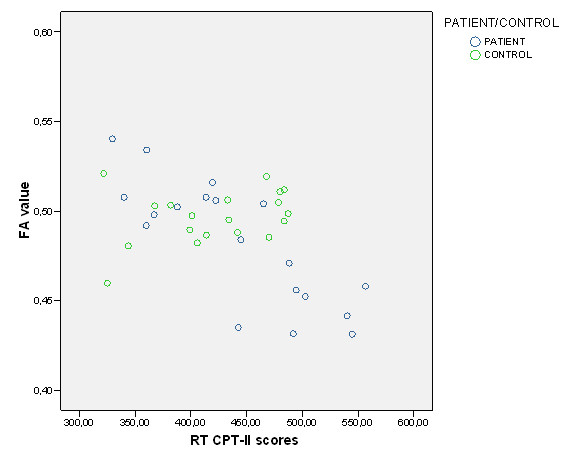
**FA values and RT CPT-II scores (for both patients and controls) in -17, 26, 22 coordinate significant cluster**. Y axis FA value in -17, 26, 22 coordinate cluster. X axis RT CPT-II scores.

In the patient group, FA correlated positively with SDMT in anterior (Table 4 and Figure 1C) and posterior (Table 4 and Figure 1A and Figure 2, 3) regions of the corpus callosum. RT CPT-II correlated negatively with FA values in the anterior part of the corpus callosum (Table 4 Figure 1B and Figure 4). No significant results were found in the control group or in the inverse contrast. No significant results where found between groups in FA values (significant coordinates from correlation analysis). The correlation analysis did not show significant results in the other variables.

**Table 4:** Anatomical regions, where FA correlated with cognitive performance in the MetSd group

Cognitive test	Corpus Callosum region	Lobe	Size	MNI coordinates			p value	r
				**x**	**y**	**z**	**(corr)**	
SDMT	Right Anterior	F	8419	21	29	28	0.016	0.8
	Left Posterior	0-T	11331	-33	-44	9	0.015	0.7
CPT-II RT								
	Left Anterior	F	25171	-17	26	22	0.003	0.7

SDMT = Symbol Digit Modalities Test; CPT-II = Continuous Performance Test II; RT = Reaction time; F = Frontal; O = Occipital; T = Temporal; Size = Cluster size at p 0.05; corr = Corrected; r = Pearson coefficient.

Age is a well known variable related to processing speed, to control the possible effect of this variable in the correlation analysis within each group, we performed a partial correlation analysis between the mean FA value for each significant cluster and the processing speed measures. We obtained significant results in correlation analysis between SDMT and mean FA measures in the significant clusters, whilst the correlation between mean FA measure and CPT II RT show a tendency in the significant cluster (Table 5).

**Table 5:** Partial Correlation analysis between mean FA (within the significant clusters) and cognitive performance in the MetSd group, controlling age effect

Cognitive test	Corpus Callosum region	MNI coordinates			p value	r
		**x**	**y**	**z**		
SDMT	Right Anterior	21	29	28	0.002	0.7
	Left Posterior	-33	-44	9	0.002	0.7
CPT-II RT						
	Left Anterior	-17	26	22	0.051	0.4

SDMT = Symbol Digit Modalities Test; CPT-II = Continuous Performance Test II; RT = Reaction time; F = Frontal; O = Occipital; T = Temporal; r = Pearson coefficient.

## Discussion

Previous studies have reported relationships between MetSd and slower processing [15,16] and subtle microstructural alterations in WM [10]. In the present study, there were more slow participants in MetSd group than in controls group, and their performance correlated with the FA values obtained by DTI. The correlations were especially significant in the corpus callosum fibers of the frontal lobe.

In a study of diabetic patients (type I) Kodl et al. (2009) [35] reported an association between FA measures in the corpus callosum splenium, corona radiata and optic radiation with time taken to copy Rey's figure and Grooved Pegboard Test scores. These results indicated a specific effect of a vascular risk factor in regions involved in processing speed. A recent functional MRI study of the oral version of the SDMT reported the involvement of anterior and posterior brain areas in the task, specifically the occipital lobes (related to the perception of visual attention/scanning) and the fronto-parietal network. The authors associated these latter areas with processing speed, attention and working memory, all processes involved in SDMT performance [36]. Similarly, Madden et al. (2009) [37] showed that correlations between FA and RT of several behavioral measures were not entirely dependent on perceptual-motor processing and may reflect deficits in decision-level processing associated with age-related decline in WM integrity within specific regions of the fronto-parietal network (central genu and splenium-parietal fasciculi).

Similarly, the anterior-posterior WM involvement in our results may also reflect the influence of these regions in attention and executive processing in the context of visuomotor control and response regulation, especially in SDMT and RT CPT-II tasks in which these processes are more directly involved. In fact, greater callosal integrity allows faster task performance, as a result of better signal transduction.

Mental slowness also correlated with extended atrophy in the corpus callosum in normal aging [38] and in subjects with WMH [21], reflecting the relationship between mental slowness and subcortical white matter damage.

Recent studies in the elderly corroborate our neuropsychological and DTI findings, showing associations between WM degeneration and slower processing speed and executive deficits, specifically in the frontal lobe [17]. Madden et al (2004) [39] also emphasized the importance of anterior and posterior corpus callosum in processing speed performance in an adult sample; in younger adults the best predictor of RT was FA values in the splenium of the corpus callosum, while in the elderly it was FA in the anterior limb of the internal capsule.

The studies reported above showed a relation between the corpus callosum and processing speed and attention in the elderly. Although most of these studies controlled the history of cardiovascular pathology and the effects of certain vascular risk factors (hypertension, presence of WMH, diabetes mellitus), they did not control the presence of other risk factors included in the MetSd diagnosis such as obesity, insulin resistance and dyslipidemia. Despite the fact that the relation between processing speed measures and FA are influenced by age, our results suggest that this relation between processing speed deficits and WM alterations in the elderly could be mediated in some way by the effect of MetSd; since this relationship was found only in our MetSd patients, and it was maintained after the post-hoc analysis controlling the possible age effect. Although the specific mechanism underlying this relationship is not clear, small-vessel disease may be responsible for the microstructural alteration in these patients due to the chronic state of vascular dysregulation in the brain [40] and blood brain barrier failure [41]. Some authors have suggested this relationship between vascular risk factors and pathological pathways such as ischemic cerebral small-vessel disease, as well as a toxic effect on neurons caused by the accumulation of advanced glycation end products and their influence on neurodegenerative mechanisms. Therefore, vascular risk factors included in MetSd may influence both cerebrovascular pathology and neurodegenerative processes [42].

Vascular risk factors are very common in aging, but when they are present in MetSd they may predispose to pathological processes and produce a very early state of deterioration, which makes successful aging more difficult [43].

DTI is a technique that allows early detection of WM damage, sometimes even in regions that appear normal on conventional anatomical images. The present study shows that the relationship between DTI measures and cognitive performance allows detection of structural and functional deficits at an initial state of deterioration, when patients are prodromal for mild vascular cognitive impairment. To the best of our knowledge, our results are the first evidence of vascular involvement in the neuropsychological performance of MetSd patients detected by means of neuroimaging.

Although our findings are suggestive, the study is limited in several ways. The most important limitation is the sample size, due to the high comorbidity of MetSd and aging with some of the exclusion criteria such as vascular pathology. Moreover, the increase of sample size as well as the inclusion of a control group with one or two vascular risk factors in future work would facilitate the analysis of the synergic effects of the vascular risk factors that comprise MetSd. In addition, as our design is cross-sectional, follow-up studies of these patients may show how the profile of cognitive impairment and brain damage evolves using longitudinal designs.

In conclusion, our study found correlations between microstructural WM changes and processing speed deficits in MetSd patients using DTI. These results suggest the existence of a relation between the presence of vascular risk factors and cognitive performance in MetSd patients. Although other factors contribute to pathological aging, MetSd in normal aging may be involved in these processes and may increase the vulnerability to pathology.

The prevalence of MetSd is increasing in modern-day society and its effect is particularly serious in the elderly. Our results highlight the importance of preventing and controlling MetSd because of its association with structural and functional damage in the WM during aging.

## Conclusion

We found significant correlations between WM alterations and cognitive impairment of MetSd patients, especially in the frontal lobe. These findings highlight the importance of MetSd prevention and control due to its association with structural and functional damage in the WM.

## Competing interests

The authors declare that they have no competing interests.

## Authors' contributions

**BS **contributed to the acquisition of subjects and DTI data, analysis, interpretation of data and preparation of manuscript. **MAJ **contributed to the study concept and design, analysis and interpretation of data, and preparation of manuscript. **NF **contributed to the acquisition of subjects and interpretation of data and preparation of manuscript. **NB**: contributed to the analyses and quantification of WM hyperintensities interpretation of data and preparation of manuscript. **CJ **contributed to the study concept and design, analysis and interpretation of data, and preparation of manuscript. **AA **contributed to the interpretation of data, and preparation of manuscript.

All authors read and approved the final manuscript.

## Pre-publication history

The pre-publication history for this paper can be accessed here:

http://www.biomedcentral.com/1471-2377/10/64/prepub
